# Case Report of acupotomy release combined with manual release under anesthesia for adhesions after unilateral total knee arthroplasty in a patient with hemophilia A

**DOI:** 10.3389/fsurg.2025.1712543

**Published:** 2025-11-17

**Authors:** Wei Wang, Xiaochan Xu, Kui Sun, Tianyao Wang, Yonghui Yang, Sanbing Wu

**Affiliations:** Department of Emergency, Second Affiliated Hospital of Anhui University of Traditional Chinese Medicine, Hefei, China

**Keywords:** hemophilia A, total knee arthroplasty (TKA), jointAdhesions, acupotomy release, manual release, coagulation factor replacement therapy

## Abstract

Patients with end-stage hemophilia often require total knee arthroplasty (TKA); however, the incidence of postoperative joint adhesions in these patients is significantly higher than that in the general population. Furthermore, due to their abnormal coagulation function, the bleeding risk associated with traditional open adhesiolysis is also higher in this patient cohort compared with the general population, and conservative rehabilitation therapy yields suboptimal outcomes. Thus, there is an urgent clinical need for a safe and effective intervention regimen. This study reports a 25-year-old male patient with severe hemophilia A (factor Ⅷ deficiency), whose baseline factor Ⅷ activity was 0.8%. Severe knee joint adhesions occurred 3 months after TKA, manifested as difficulty in walking, sitting, and standing. A combined treatment regimen of “perioperative precise coagulation factor replacement + ultrasound-guided acupotomy release under anesthesia + graded manual release” was adopted for the intervention of post-TKA adhesions in patients with severe hemophilia A. The results showed that this combined regimen could achieve precise release while reducing the risk of bleeding, significantly improve joint function, and provide a new diagnostic and therapeutic strategy for the clinical treatment of such diseases.

## 1 Introduction

Hemophilic arthropathy is a chronic progressive disorder caused by coagulation factor deficiency. Among hemophilias, hemophilia A (factor Ⅷ deficiency) accounts for approximately 80%–85% (1). Patients with severe hemophilia A are more likely to progress to end-stage joint disease due to repeated joint bleeding (2). TKA is a core surgical method to improve the quality of life of such patients, but the incidence of postoperative joint adhesions in patients with severe hemophilia A is significantly increased. The pathogenesis is closely related to incomplete synovectomy during surgery, fibrin deposition due to repeated postoperative microbleeding, and scar tissue hyperplasia caused by delayed rehabilitation (3). Joint adhesions not only limit joint movement but also increase intra-articular pressure, further inducing hemorrhagic synovitis and forming a vicious circle of “adhesion-bleeding-re-adhesion” (4).

In traditional treatment regimens, simple manual release leads to poor patient compliance due to severe pain, and the release force is difficult to control, which easily causes intra-articular bleeding. Although open lysis of adhesions can completely remove adhesions, it causes great surgical trauma. The perioperative bleeding risk of patients with severe hemophilia A is as high as 35%–40% (5), and the postoperative infection rate is also significantly higher than that of the general population and patients with mild hemophilia (6). Therefore, exploring a minimally invasive, precise, and low-risk release technology is the key to solving post-TKA adhesions in patients with severe hemophilia A.

Recent studies have shown that closed release under anesthesia can break through the adhesion threshold with the help of muscle relaxation, reducing pain stress in patients. However, this technique has a limited release effect on deep fibrous adhesions, and the improvement range of postoperative range of motion (ROM) is only 30°–40° (7). As a minimally invasive technique of traditional Chinese medicine, acupotomy release has the advantages of precise cutting of fibrous scars and minimal trauma. It has been used in the treatment of post-TKA adhesions in the general population, with a postoperative complication rate of less than 5% (8). However, there are no reports on the safety and efficacy of acupotomy release in patients with severe hemophilia A.

In this study, “acupotomy release under anesthesia + manual release” was combined for the treatment of post-TKA adhesions in patients with severe hemophilia A: the deep adhesions were precisely cut by acupotomy to reduce the force required for manual release, thereby reducing the risk of bleeding; aiming at the extremely low baseline coagulation factor activity of severe patients, a coagulation management regimen of “preoperative high-dose enhancement + intraoperative dynamic monitoring + postoperative stepwise reduction” was adopted to ensure perioperative coagulation safety; before and after treatment, multi-dimensional scores such as Hemophilia Joint Health Score (HJHS), Hospital for Special Surgery Knee Score (HSS), and Knee Society Score (KSS), combined with imaging examinations and gait analysis, were used to comprehensively quantify the treatment effect, providing evidence-based basis for the clinical promotion of this regimen. This study aims to find a better clinical regimen for the treatment of post-TKA joint adhesions in patients with severe hemophilia A, so as to improve the symptoms of joint adhesions and enhance their quality of life.

## 2 Case data

The patient was a 25-year-old male, with a height of 172 cm, a weight of 68 kg, and a body mass index (BMI) of 23.0 kg/m^2^. He was diagnosed with severe hemophilia A 8 years ago, with a baseline factor Ⅷ activity of 0.8% (normal reference range: 50%–150%). He regularly received recombinant human coagulation factor Ⅷ infusion (3 times a week, 250 IU/kg each time), with no history of transfusion allergy or cardiovascular disease.

Three years ago, due to severe hemophilic arthritis of the right knee (Kellgren-Lawrence grade Ⅳ), the patient underwent right TKA at the First Affiliated Hospital of Anhui Medical University.Preoperatively, he received factor Ⅷ augmentation therapy to increase his factor Ⅷ activity to 100%. Intraoperative blood loss was approximately 250 mL. Postoperatively, low-molecular-weight heparin was routinely used for anticoagulation for 14 days. After 6 weeks of rehabilitation training, the knee ROM reached 0°–115°, and he could walk independently.

Three months postoperatively, the patient developed right knee stiffness without an obvious cause, accompanied by a gradual decrease in range of motion (ROM) and pain during walking.He needed crutches to assist walking, and required assistance to sit down and stand up, taking about 3 min. The patient had previously undergone physical therapy, including ultrasound therapy, wax therapy, and continuous passive motion (CPM) training, for 3 months in another hospital, but the symptoms did not improve. During the training, 2 episodes of mild joint bleeding were induced by rehabilitation training, so he was admitted to the Second Affiliated Hospital of Anhui University of Chinese Medicine.

After admission, the research team evaluated the patient's knee function, including HJHS, HSS, KSS, and VAS (Table 1). The results of knee examination showed that the active extension ROM of the right knee was 20° (Supplementary Figure S1), the active flexion ROM was 70° (Supplementary Figure S2), the passive extension ROM was 30°, and the passive flexion ROM was 100°; the flexion contracture degree was 25°; varus-valgus stress test (−), drawer test (−); patellar mobility: 1 cm inward movement, 0.8 cm outward movement (2.5 cm inward movement, 2.2 cm outward movement on the healthy side). The results of Manual Muscle Testing (MMT) showed that the quadriceps muscle strength was grade 3, the hamstring muscle strength was grade 4, and the ankle dorsiflexion muscle strength was grade 4; due to long-term limited activity, the circumference of the affected thigh was 1.5 cm thinner than that of the healthy side.

**Table 1:** Results of functional scores and scale evaluation.

Evaluation index	Preoperative (Before treatment)	1 week postoperative	1 month postoperative	3 months postoperative	Normal reference value
Hemophilia Joint Health Score (HJHS)	28 points	32 points	34 points	38 points	0–10 points (mild impairment)
Hospital for Special Surgery Knee Score (HSS)	52 points	68 points	78 points	86 points	≥85 points (excellent/good)
Knee Society Score (KSS)	60 points	70 points	75 points	88 points	≥80 points (excellent/good)
Timed Up and Go Test (TUG)	15 s	13 s	12.3 s	9 s	<10 s (normal)
Visual Analogue Scale (VAS)	5 points	2 points	1 point	0 points	0 points (no pain)

**Figure 1:**
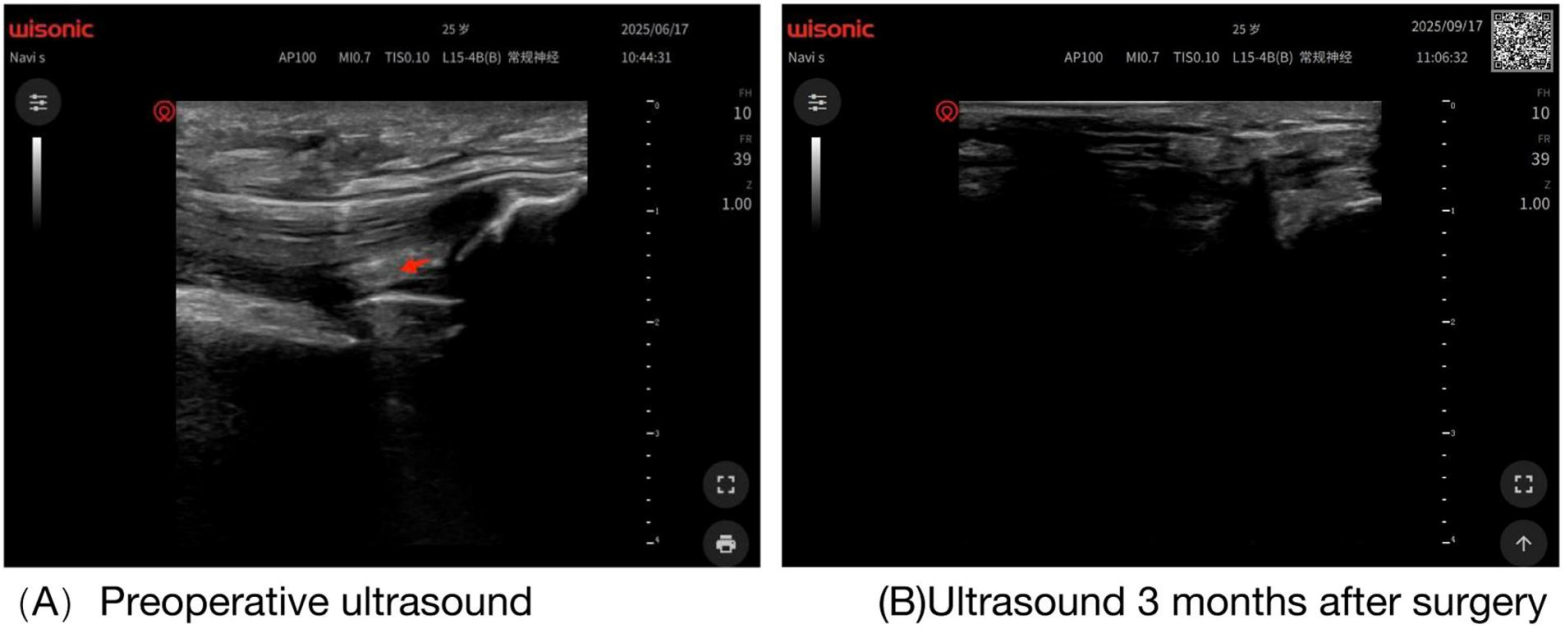
**(A)** Preoperative knee ultrasound image (red arrow indicates adhesion site). **(B)** Knee ultrasound image three months postoperatively.

Knee x-ray showed that the prosthesis was in a good position without loosening or subsidence, no obvious narrowing of the joint space, and a small amount of soft tissue swelling in the suprapatellar bursa (Supplementary Figure S3). Ultrasound examination showed that strip-like hyperecho was observed in the patellofemoral joint space, suggesting the formation of fibrous adhesions; the synovium in the medial space of the tibiofemoral joint was thickened, accompanied by a small amount of fluid dark area; the echo at the attachment of the quadriceps tendon to the patella was uneven, suggesting local scar adhesion (Figure 1). In addition, the results of preoperative gait evaluation and Timed Up and Go Test (TUG) showed that the patient had obvious claudication, and the affected knee presented a “stiff gait” (small flexion-extension range) when walking (Supplementary Figure S4), with a TUG time of 15 s (Supplementary Figure S9).

## 3 Treatment process

### 3.1 Perioperative coagulation management

Aiming at the extremely low baseline coagulation factor activity of the patient with severe hemophilia A, the research team adopted a coagulation management strategy of “high-dose enhancement + dynamic monitoring + stepwise reduction”: 24 h before the procedure: Recombinant human coagulation factor Ⅷ was intravenously infused at a dose of 40 IU/kg to increase the patient's factor Ⅷ activity to 80%–100%; 2 h before surgery: Additional infusion of 25 IU/kg to ensure that the factor Ⅷ activity of the patient was ≥90% when entering the operating room as patients with severe hemophilia require higher factor activity preoperatively to reduce bleeding risk during puncture and the procedure;Factor Ⅷ activity was monitored every 1.5 h. If the activity was lower than 80%, an additional 15 IU/kg was infused to maintain the activity at 85%–100% throughout the operation; 0–72 h after surgery: Infusion of 25 IU/kg every 10 h to maintain the activity at 80%–100%; 4–7 days after surgery: Daily infusion of 20 IU/kg to maintain the activity at 60%–80%; 8–14 days after surgery: Infusion of 15 IU/kg every 24 h to maintain the activity at 50%–60%; 15–21 days after surgery: Infusion of 10 IU/kg every 48 h, gradually transitioning to the routine maintenance dose 250 IU/kg, 3 times a week.

During the treatment period, the patient's factor Ⅷ activity, Prothrombin Time (PT), Activated Partial Thromboplastin Time (APTT), and hemoglobin level were detected daily; knee ultrasound was rechecked 3 days and 7 days after surgery to dynamically observe the presence of intra-articular bleeding or increased effusion.

### 3.2 Anesthesia and release operation

#### 3.2.1 Anesthesia regimen

The patient underwent “local infiltration anesthesia combined with intravenous sedation” during the procedure: 20 mL of 0.5% lidocaine containing 1:200,000 epinephrine was injected around the knee joint to reduce local bleeding and block the peripheral nerves of the joint; Propofol (1.5 mg/kg) was intravenously administered—at a lower dose than that for conventional patients to minimize circulatory fluctuations—and remifentanil (0.4 μg/kg) was used to maintain sedation while preserving the patient's spontaneous breathing. During the operation, heart rate, blood pressure, blood oxygen saturation, and Bispectral Index were continuously monitored, and the bleeding situation in the surgical field was recorded every 5 min.

#### 3.2.2 Ultrasound-guided acupotomy release

**Figure 2:**
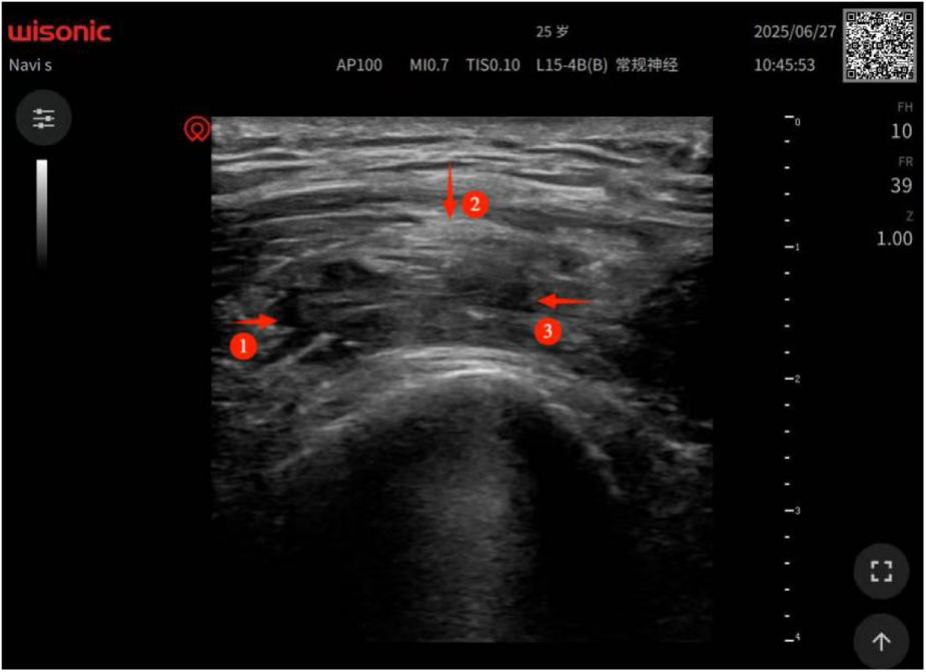
Preoperative ultrasound image: three main adhesive regions (indicated by three red arrows): ① adhesion at the attachment site of the patellar tendon to the tibial tuberosity; ② adhesion between the quadriceps tendon and the superior pole of the patella, showing uneven echo and poor mobility; ③ synovial-capsular adhesion in the medial tibiofemoral joint space accompanied by a sonolucent area, which is considered to be associated with old bleeding.

Before acupotomy treatment, the adhesion targets were first located by ultrasound, and 3 main adhesion areas were clearly identified (Figure 2): ① the attachment of the patellar tendon to the tibial tuberosity; ② the quadriceps tendon and the superior pole of the patella uneven echo at the adhesion site, poor mobility; ③ the synovium-joint capsule in the medial space of the tibiofemoral joint accompanied by fluid dark area, considered as adhesion related to old bleeding.

**Figure 3:**
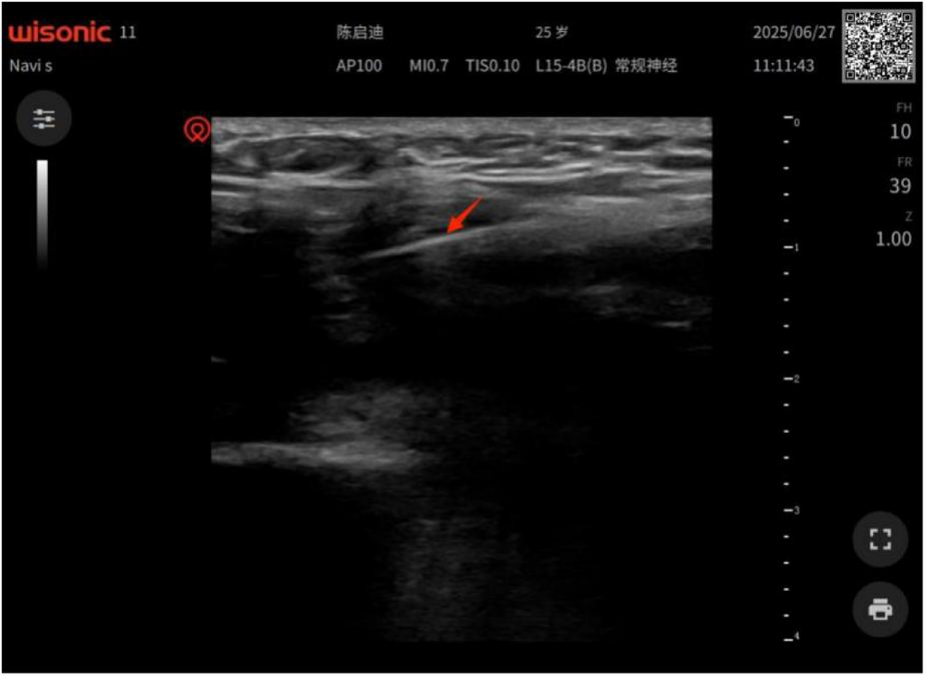
Under ultrasound: a strong echo of the needle tip is visible when the needle tip reaches the surface of the adhesive tissue.

After positioning, routine disinfection and draping were performed. The course of the femoral artery and saphenous nerve was confirmed under ultrasound guidance. After avoiding the vascular nerve bundle, a disposable sterile acupotomy 2 mm × 50 mm was selected and inserted vertically into the skin along the direction of the tendon. When the needle tip reached the surface of the adhesion tissue, “needle tip hyperecho” was observed under ultrasound, and the depth of the acupotomy was fixed to avoid damage to the prosthesis or intra-articular structures (Figure 3).

The specific acupotomy release operation was as follows: ① Around the patellar tendon: The acupotomy was used for “longitudinal cutting” 3–4 times parallel to the direction of the patellar tendon, with a cutting depth of <5 mm each time. During the cutting process, the rupture of adhesions was observed in real time by ultrasound until the mobility of the patellar tendon was increased by 50% compared with that before the operation; ② Quadriceps tendon: The “fan-shaped stripping” technique was used to release the adhesion between the tendon and the patella, and vertical cutting was avoided to prevent tendon rupture, as the tendon repair ability of severe patients is poor; ③ Joint capsule: The acupotomy was slowly inserted into the joint capsule for “point-like release”, and about 3 mL of old effusion in the joint was released sent for routine and biochemical examination to rule out infection. After confirming the disappearance of effusion by ultrasound, the acupotomy was withdrawn (Supplementary Figure S10).

After the release of each target, pressure hemostasis was performed for 8 min. After confirming no active bleeding, sterile dressing was used for pressure bandaging. The total operation time was about 30 min longer than that for conventional patients to ensure slow operation and reduce bleeding, with an intraoperative blood loss of <8 mL.

#### 3.2.3 Graded manual release

**Figure 4:**
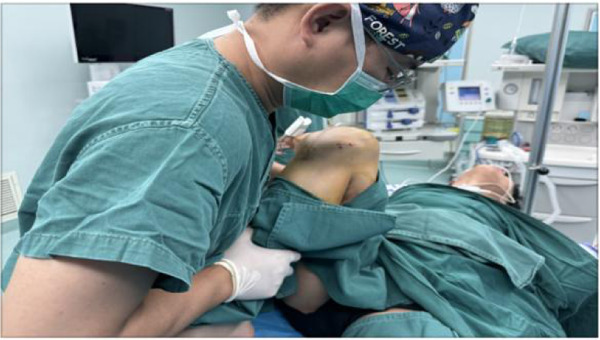
Extension correction operation: the patient was in the supine position. The operator placed one hand above the patella (mild pressure to avoid patellar dislocation) and held the proximal end of the lower leg with the other hand, slowly pressing down the lower leg to correct the flexion contracture. The extension position was maintained for 45 s, repeated 3 times, and finally, the knee joint achieved 0° extension without contracture.

After the completion of acupotomy release, “low-force graded manual release” was performed under the state of anesthetic muscle relaxation the release force was reduced according to the fragile tissue characteristics of severe patients (Supplementary Figure S11), and the specific steps were as follows (9):

Preliminary Loosening: The operator held the knee joint with both hands, with the thumbs placed on both sides of the patella and the other fingers encircling the lower leg. The knee joint was slowly passively flexed and extended 5 times the angle was gradually increased from 30° to 100°, with an increase range of <10° each time to relieve superficial adhesions and avoid sudden force;

Flexion Release: The assistant fixed the patient's thigh to avoid increased joint stress caused by thigh shaking, and the operator held the distal end of the lower leg with both hands palms upward to reduce tibial stress, slowly increasing the knee flexion angle. When resistance was encountered, the position was maintained for 45 s the static stretching time was longer than that for conventional patients. After the resistance was reduced, the angle was continued to be increased until the flexion reached 125°. Ultrasound confirmed that there was no obvious resistance in the tibiofemoral joint space and no abnormal bleeding;

Extension Correction: The patient was in the supine position. The operator placed one hand above the patella mild pressure to avoid patellar dislocation and held the proximal end of the lower leg with the other hand, slowly pressing down the lower leg to correct the flexion contracture. The extension position was maintained for 45 s, repeated 3 times, and finally, the knee joint achieved 0° extension without contracture (Figure 4). During the operation, knee hyperextension was avoided to prevent ligament injury;

Patellar Movement: The patella was pinched with the thumb and index finger moderate force to avoid skin injury and pushed in the up-down and inside-out directions until the patellar mobility was restored to 80% of that of the healthy side 2.0 cm inward movement, 1.8 cm outward movement. During the movement, the presence of bleeding around the patella was observed in real time (Supplementary Figure S5).

### 3.3 Postoperative rehabilitation training

Aiming at the characteristics of easy bleeding and slow tissue repair in patients with severe hemophilia A, a rehabilitation strategy of “slow start, low intensity, and gradual transition” was adopted:

#### 3.3.1 Acute phase management

Immediate ice application: Within 72 h after surgery, ice was applied for 25 min per session, six times daily prolonged ice application time to reduce swelling and bleeding risk; Passive ROM training: Initiation was delayed until 48 h postoperatively to avoid bleeding induced by early activity; Muscle strength training: Isometric contraction training of the quadriceps was started 3 days after surgery contraction for 15 s each time, 10 times per group, 4 groups per day, with reduced contraction intensity, and active knee flexion and weight-bearing training were avoided.

#### 3.3.2 Recovery phase management

Active ROM training: Started 2 weeks after surgery delayed by 1 week compared with conventional patients, combined with balance training single-leg standing, 20 s each time, 3 groups per day, gradually increasing the time; Gait training: Started 4 weeks after surgery delayed crutch removal to ensure coagulation stability. Under the guidance of a rehabilitation therapist, the patient first walked with a single crutch, then transitioned to walking without assistance 2 weeks later to correct claudication;Functional training: Sit-to-stand training was started 6 weeks after surgery gradually transitioning from high chairs to medium chairs to low chairs, with an interval of 30 min between each training session to avoid fatigue and stair-climbing training 1 step each time, holding the handrail with both hands, gradually increasing to 2 steps.

### 3.4 Postoperative follow-up results

The patient was followed up and evaluated within 3 months after surgery, and the results were as follows:Functional Scores: HJHS increased from 28 points severe impairment before surgery to 38 points mild impairment 3 months after surgery, among which the scores of “pain”, “mobility”, and “swelling” improved most significantly decreasing from 6 points to 2 points, 5 points to 1 point, and 4 points to 0 points, respectively, indicating that the patient's joint inflammatory response was significantly relieved after treatment, and there was no score deterioration caused by new bleeding (10); the HSS score reached 86 points, an increase of 34 points compared with that before surgery, among which the “mobility” increasing from 8 points to 20 points and “function” increasing from 12 points to 25 points improved significantly; the VAS score decreased to 0 points, and no joint bleeding occurred during the follow-up period; the KSS reached 88 points, and the knee function improved from “poor” 40 points to “excellent” 85 points, with good prosthesis stability and no loosening or instability. TUG Test: The time was shortened to 9 s (Supplementary Figure S12) 3 months after surgery. The patient did not need auxiliary tools, with smooth movements and no pauses or pain, indicating that the independent walking and transfer abilities were fully restored, and continued to improve compared with 1 month after surgery 12.3 s, which showed that the rehabilitation strategy was gradual and did not induce complications due to overtraining (Table 1). Knee ROM: It reached 0°–110° 1 week after surgery, 0°–120° 1 month after surgery, and 0°–125° 3 months after surgery. The flexion contracture degree was completely corrected from 25° to 0°, and no ROM regression was observed in the rechecks at all time points (Supplementary Figure S6). Patellar Mobility: 3 months after surgery, the patella moved 2.2 cm inward and 1.9 cm outward, close to the level of the healthy side. No performance new adhesions around the patella (Supplementary Figure S7). Muscle Strength and Muscle Morphology: 3 months after surgery, the quadriceps muscle strength recovered to grade 4+, the hamstring and ankle muscle strength reached grade 5; the circumference of the affected thigh was 0.5 cm thinner than that of the healthy side improved by 1 cm compared with before surgery, indicating that muscle atrophy was gradually recovered and not aggravated by long-term immobilization.3 months after surgery, the stance phase time of the affected side was 0.62 s, the step length was 58 cm (60 cm on the healthy side), and the maximum knee flexion angle was 105°. The gait was symmetrical, with no claudication or “stiff gait”, and the knee joint flexed and extended naturally when walking, showing significant improvement compared with before surgery (Supplementary Figure S8).

## 4 Discussion

Compared with patients with mild-to-moderate hemophilia, those with severe hemophilia A have a higher perioperative bleeding risk; thus, a “high-dose, short-interval” coagulation factor replacement regimen is required. In this study, the patient's preoperative factor Ⅷ activity was maintained at ≥90%. During the procedure, factor Ⅷ activity was monitored every 1.5 h, and factor Ⅷ was infused every 10 h in the early postoperative period. This regimen was more intensive than the conventional regimen, and finally, the intraoperative blood loss was <8 mL and no postoperative bleeding occurred, which was consistent with the coagulation management conclusion of guideline (11). For minimally invasive treatment of severe hemophilia maintaining factor activity ≥80% can reduce the bleeding rate to less than 2%.

Aiming at the characteristics of fragile tissues and poor repair ability of severe patients, this case adopted the operation method of “ultrasound real-time guidance + low-force cutting + prolonged compression time”: the acupotomy cutting depth was <5 mm, and the manual release force was <40 N lower than 50 N for conventional patients (12), to avoid excessive injury causing bleeding or tendon rupture; at the same time, the postoperative rehabilitation start time was delayed to 48 h to reduce the bleeding risk induced by early activity, which was consistent with the recommendation of “slow start of postoperative rehabilitation for severe patients” in the WFH Guidelines for the Management of Hemophilia, 3rd edition (13).

Previous studies on post-TKA adhesions in the general population have shown that the improvement range of ROM 3 months after manual release under anesthesia is about 40° (7), while the improvement range of ROM in patients with severe hemophilia A in this study reached 55° from 90° to 145°, and the HJHS score improved by 10 points (10). This may be because acupotomy release solved the deep adhesions, and the comprehensive coagulation management did not affect the recovery due to bleeding. In addition, the TUG time 3 months after surgery in this study was better than the release result of the general population reported by Louis et al. (10), suggesting that the joint function of patients with severe hemophilia A can reach a level close to that of the general population after precise treatment.

The complication rate of traditional open lysis of adhesions in patients with severe hemophilia A is as high as 35%–40% (5), but no complications (e.g., bleeding, infection) were observed in the patient during the study period. The total amount of coagulation factor used within 21 days after surgery was 420 IU/kg, which was lower than 600–800 IU/kg of open surgery (14), significantly reducing the treatment cost and transfusion-related risks such as inhibitor production. At the same time, this study adopted local infiltration anesthesia + intravenous sedation, avoiding the impact of general anesthesia on the circulatory system of severe patients, and further improving the treatment safety.

The treatment of post-TKA adhesions in patients with severe hemophilia A needs to take both “release effect” and “coagulation safety” into account, and a differentiated regimen should be formulated according to the patient's baseline coagulation factor activity and the degree of joint adhesion: ① Coagulation management: For patients with baseline activity <1%, the preoperative activity should be ≥90%, and the postoperative early infusion interval should be ≤10 h; ② Release operation: Ultrasound guidance must be used to avoid blind cutting; ③ Rehabilitation training: Prolong the acute phase, gradually increase the training intensity, and avoid inducing bleeding.

Ultrasound plays an irreplaceable role in the treatment of severe patients: ① Preoperative positioning of adhesions, blood vessels, and nerves to reduce operation risks; ② Intraoperative real-time monitoring of release effect and bleeding situation, adjusting the operation in a timely manner; ③ Postoperative dynamic evaluation of adhesion recurrence and effusion, early detection of complications. Compared with Magnetic Resonance Imaging (MRI), ultrasound has the advantages of convenience and economy, and is more suitable as an imaging method for long-term postoperative follow-up of patients with severe hemophilia (15).

Future research directions include: ① Exploring the standardized operation process of “ultrasound-guided acupotomy release” for patients with severe hemophilia A (such as the number of targets, cutting depth, and release force); ② Carrying out postoperative precise rehabilitation combined with rehabilitation robots to further reduce the bleeding risk through quantitative force control; ③ Conducting randomized controlled trials to compare the efficacy and cost-effectiveness ratio of this combined regimen with traditional open lysis of adhesions.

## 5 Conclusion

For a 25-year-old patient with severe hemophilia A (factor Ⅷ deficiency) who developed post-TKA adhesions, the individualized regimen—comprising high-dose coagulation factor replacement, ultrasound-guided low-force acupotomy release under anesthesia, graded manual release, and slow-initiation rehabilitation—achieved precise and effective adhesion release while ensuring safety. This regimen significantly improved the patient's joint ROM, functional scores, and gait, with no complications (e.g., bleeding, infection) observed. This regimen provides a promotable minimally invasive strategy for the clinical treatment of post-TKA adhesions in patients with severe hemophilia A, and is worthy of further research and application.

## Data Availability

The raw data supporting the conclusions of this article will be made available by the authors, without undue reservation.
