# Supplementary material for: Case Report of acupotomy release combined with manual release under anesthesia for adhesions after unilateral total knee arthroplasty in a patient with hemophilia A
Source: Front Surg. 2025 Nov 17;12:1712543. doi: 10.3389/fsurg.2025.1712543 (PMC12665685; doi:10.3389/fsurg.2025.1712543)

S1. Preoperative active range of motion (ROM) of the right knee joint (extension)


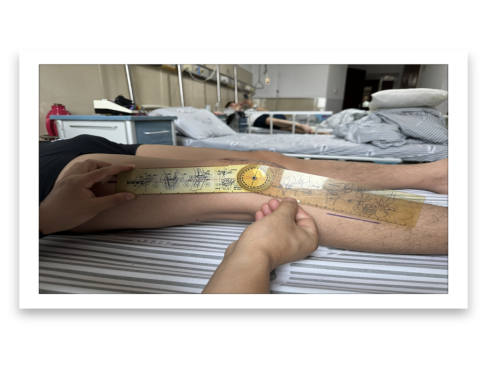


S2. Passive range of motion (ROM) of the right knee joint (flexion)


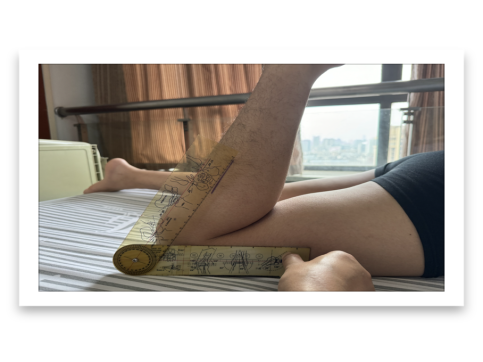


S3. Preoperative knee X-ray: The prosthesis is well-positioned without loosening or subsidence, no obvious joint space narrowing, and mild suprapatellar bursa soft tissue swelling


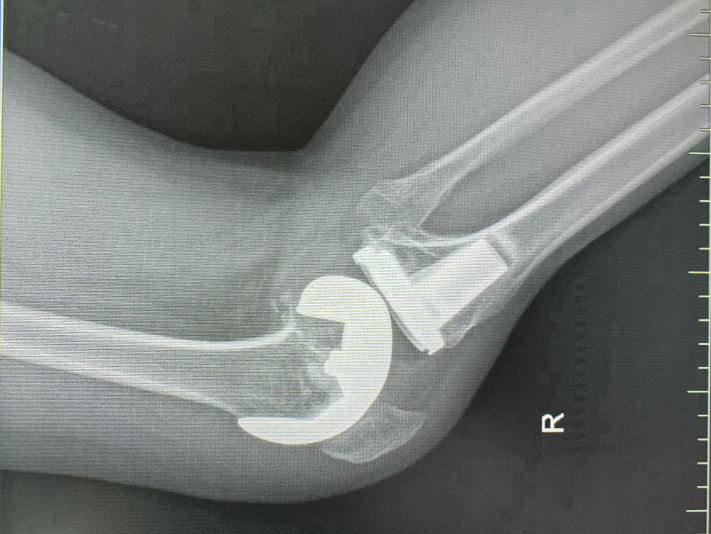


S4. Preoperative gait: The patient had obvious claudication, and the affected knee presented a "stiff gait" (small flexion-extension range) when walking


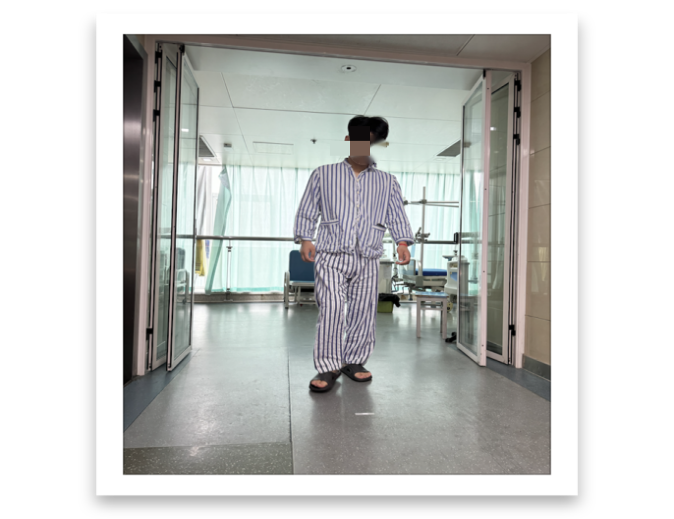


S5. Patellar movement operation: The patella was pinched with the thumb and index finger moderate force to avoid skin injury and pushed in the up-down and inside-out directions until the patellar mobility was restored to 80% of that of the healthy side


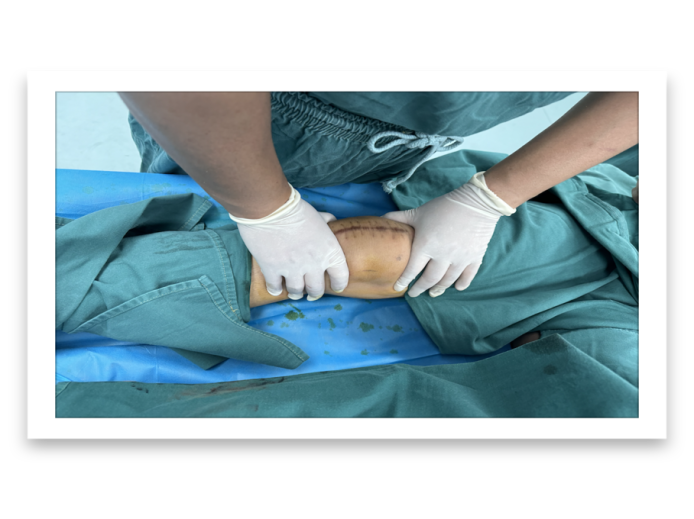


S6 Photos of knee ROM 3 months after surgery (Left: Extension position; Right: Flexion position)


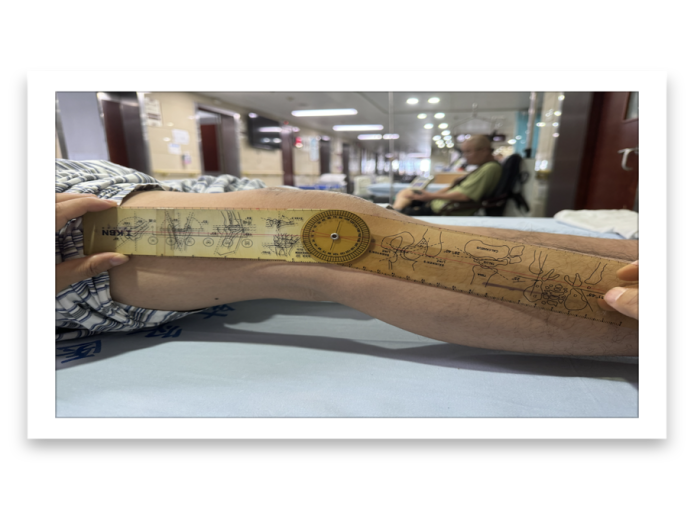

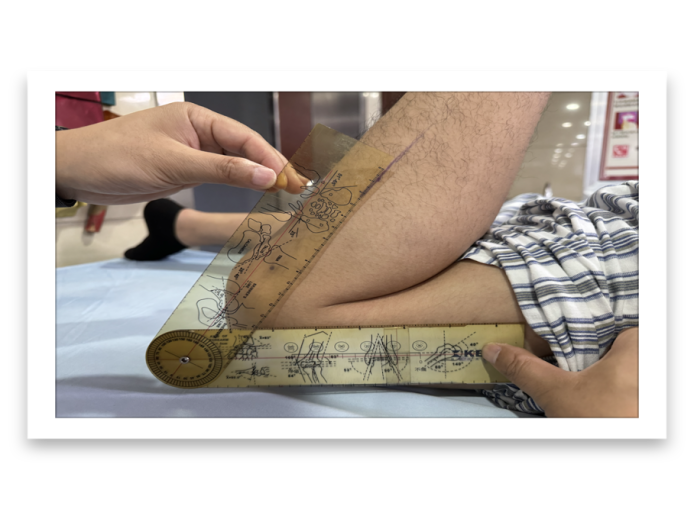


S7. Measurement results of patellar mobility 3 months after surgery: 2.2 cm inward movement, 1.9 cm outward movement


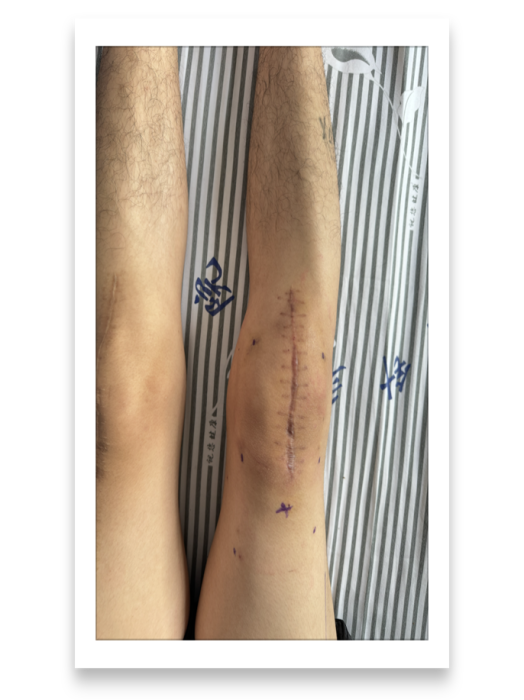


S8. Gait evaluation results 3 months after surgery: The stance phase time of the affected side was 0.62 seconds , the step length was 58 cm , the maximum knee flexion angle was 105°, the gait was symmetrical, with no claudication or "stiff gait", and the knee joint flexed and extended naturally when walking


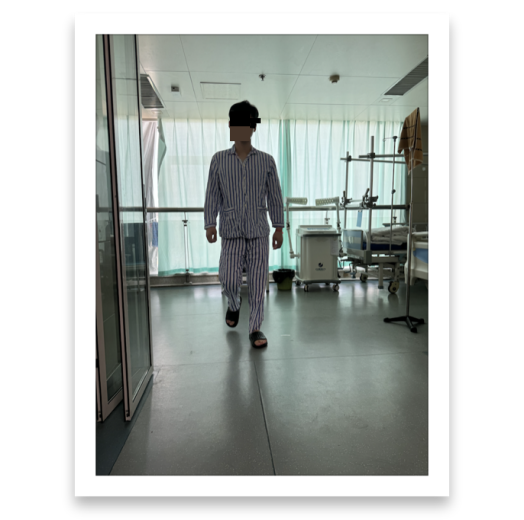

Supplement: Supplementary file 1 [file Supplementaryfile1.docx]
