# Supplementary material for: Case Report of acupotomy release combined with manual release under anesthesia for adhesions after unilateral total knee arthroplasty in a patient with hemophilia A
Source: Front Surg. 2025 Nov 17;12:1712543. doi: 10.3389/fsurg.2025.1712543 (PMC12665685; doi:10.3389/fsurg.2025.1712543)

S10.Ultrasound guided needle knife release


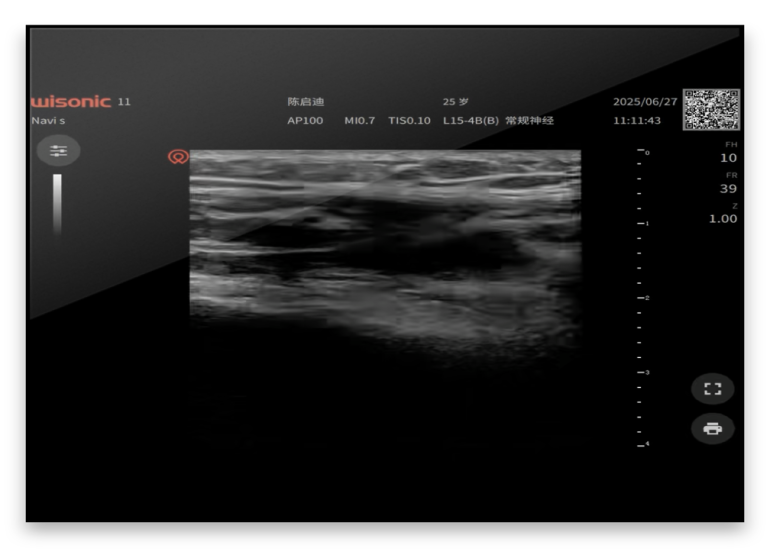


S9.Patient's preoperative Timed Up and Go Test walking video (Time 15S)


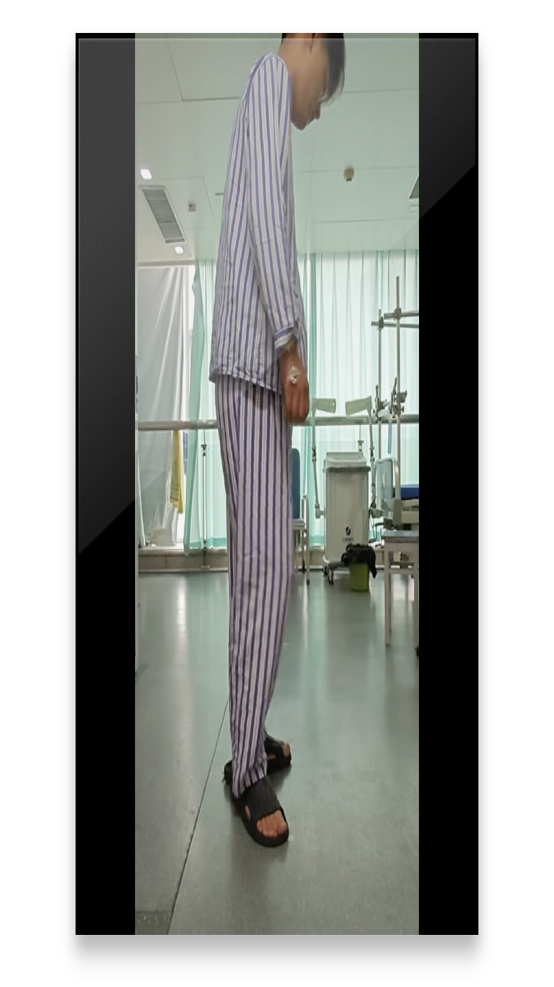


S11.Low-force graded manual release


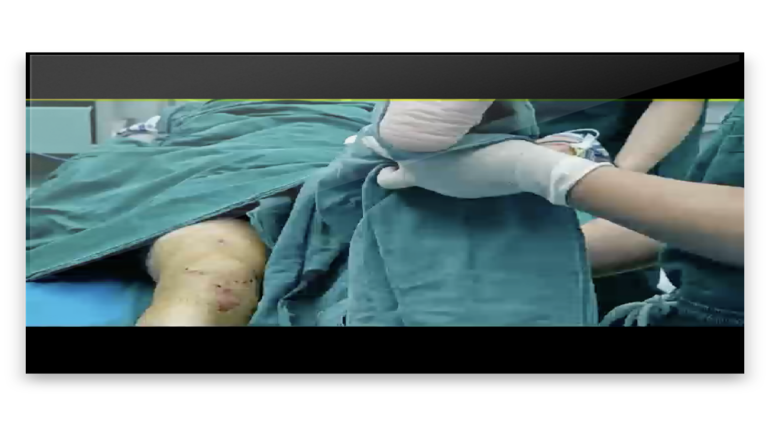


S12.Patient's 3 Months postoperative Timed Up and Go Test walking video(Time 9S)


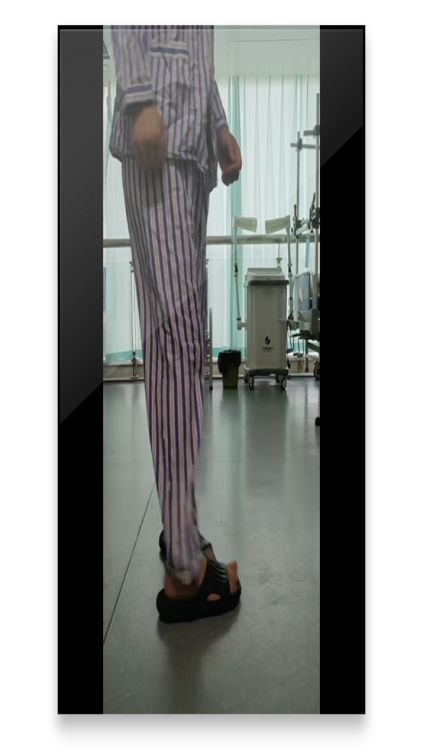

Supplement: Supplementary file 2 [file Supplementaryfile2.docx]
